# Real-world evaluation of weekly subcutaneous treatment with semaglutide in a cohort of Italian diabetic patients

**DOI:** 10.1007/s40618-022-01799-2

**Published:** 2022-04-16

**Authors:** P. Marzullo, T. Daffara, C. Mele, M. Zavattaro, A. Ferrero, M. Caputo, F. Prodam, G. Aimaretti

**Affiliations:** 1 Department of Translational Medicine, Università del Piemonte Orientale, Via Solaroli 17, 28100 Novara, Italy; 2 Endocrinology and Diabetes Unit, AOU Ospedale Maggiore Della Carità, Novara, Italy; 3 Laboratory of Metabolic Diseases, IRCCS, Istituto Auxologico Italiano, Piancavallo, Verbania Italy; 4 Department of Health Sciences, Università del Piemonte Orientale, Novara, Italy

**Keywords:** Type 2 diabetes mellitus, Obesity, Once-weekly semaglutide, GLP-1 receptor agonists

## Abstract

**Purpose:**

Registered trials and real-world evidence (RWE) studies provided evidence on the efficacy of once-weekly (OW) semaglutide on hyperglycaemia and cardiovascular risk factors as add-on or de-novo treatment in type 2 diabetes (T2D).

**Methods:**

In a retrospective analysis of electronic data files from 258 T2D patients, this RWE study aimed to explore the impact of OW semaglutide on biochemical and anthropometric outcomes after 6 and 12 months in patients receiving at least one prescription of OW semaglutide between September 2019 and May 2021.

**Results:**

During the study period, 154 and 56 consecutive patients completed the 6 and 12 months of OW semaglutide treatment. HbA1c levels decreased by -1.02±0.1% after 6 months and -1.1±0.1% after 12 months of OW semaglutide (*p*<0.0001 for both). At these time-points, HbA1c values were <7% in 61% and 57% of cases. HbA1c reduction was greater in patients with higher baseline HbA1c levels and it occurred irrespective of gender, age, insulin therapy and complications. The residual number of cases with HbA1c ≥9% by the study end was low (5.3% vs 18.9% at baseline). Weight loss occurred in 73.5% and 78.1% of cases and, compared to baseline, it was ≥5% in 21.2- 25.4% and ≥10% in 6.8-18.2% after 6 and 12 months, respectively. Significant predictors of HbA1c reduction after 6 months of OW semaglutide treatment were baseline HbA1c (*p*<0.0001), bodyweight reduction (*p*<0.0001) and disease duration (*p*<0.001), while baseline HbA1c was the only predictor of HbA1c response after 12 months (*p*<0.0001). Reported adverse events were consistent with the known safety profile of semaglutide.

**Conclusions:**

Real-world evaluation of weekly subcutaneous treatment with semaglutide in a cohort of Italian diabetic patients.

## Introduction

Incretins are peptides secreted in response to meal ingestion which, at physiological concentrations, stimulate insulin release and reduce glucagon secretion. The incretin family comprises several peptide hormones, including secretin, VIP, pituitary adenylate cyclase-activating polypeptide (PACAP), glucagon as well as glucagon-like peptides, i.e. GLP-1 and GLP-2, glycentin, oxyntomodulin and gastric inhibitory peptide (GIP) [1]. Research has progressively focused on the development of drugs eliciting the so-called incretin effect through mono-, dual- and triple agonism of these peptides [2]. Glucagon-like peptide 1 receptor agonists (GLP-1RAs) are recognized glucose-lowering agents that act through pancreatic and extra-pancreatic mechanisms, these latter including decrease of gastric motility and central control of food intake [3]. In addition to achieving glycemic targets, chronic treatment with GLP-1RAs has proved the ability to promote cardiovascular (CV) benefits and reduce the risk of death from CV disease (CVD), nonfatal myocardial infarction, or nonfatal stroke in T2D patients [4–8]. Further, treatment with GLP-1RAs benefits kidney protection through reduction of albuminuria, as well as by controlling progression of diabetic kidney disease (DKD) to end-stage renal disease (ESRD) independent of albuminuria [9]. In turn, kidney effects advantage CV health independent of glycemic control [10]. Cumulatively, these effects appear internally consistent across the drug class [11] and highlight a potential dual advantage for patients with atherosclerotic CVD (ASCVD) and DKD [12]. Moreover, their intrinsic effects on insulin and glucagon are tailored in a glucose-dependent manner, thus posing a low risk of hypoglycemia and making them one of the most effective and safest options when a more intensive antidiabetic treatment is required. Finally, the once-weekly subcutaneous administration of some GLP-1RAs helps supporting patients’ compliance and adherence to treatment. Given this wide range of benefits, GLP-1RAs are currently recommended as a second-line therapy in T2D and can be prescribed as a first-line treatment when it is necessary to enhance therapy using an injectable agent [13]. In the presence of ASCVD or high/very high cardiovascular risk (CVR), GLP1-RA can be considered as first-line treatment [14].

Semaglutide is a long-acting GLP-1 analog with a once-weekly (OW) extended release, developed to treat T2D patients who are unable to achieve their hemoglobin A1c (HbA1c) goals with other anti-hyperglycemic medications. Marketing of OW semaglutide has been granted in Europe since February 2018 and has been available for therapeutic use in Italy since September 2019 [15, 16]. The efficacy of OW semaglutide as monotherapy or as add-on therapy has been extensively investigated in the Semaglutide Unabated Sustainability in Treatment of Type 2 Diabetes (SUSTAIN) program, which comprises ten clinical trials that compared OW semaglutide to placebo or other antidiabetic treatments. Results showed a robust reduction in HbA1c up to 1.5–1.8%, a reduction in major adverse CV events (CVE) rates, kidney prevention and weight loss in patients treated with OW semaglutide as compared to placebo or other treatments [17–20]. Existing clinical trial data suggest a combined effect of OW semaglutide on the dual target of glucose and obesity control, implying a particular advantage for diabetic patients with obesity. There is a need for complementary real-world evidence to validate clinical trial results in a wider pool of patient types and for specific countries or regions [21]. So, to complement data generated by the SUSTAIN program, an analysis conducted on standard users of OW semaglutide with broad representativeness of the routine patient population referred to the clinical practice could support with real-world evidence the effectiveness of OW semaglutide and provide insights on response predictors. The purpose of this observational study was to expand the knowledge on OW semaglutide treatment in a real-world setting and to provide guidance on its efficacy on metabolic, anthropometric and hepato-renal outcomes in patients with T2D on other previous treatment schedules consecutively enrolled for up to 12 months. Change in HbA1c and HbA1c target attainment between pre- and post-index measurements were primary study aims. Secondary study aims included changes in bodyweight and kidney function tests, as well as analysis of interactions between HbA1c and predictors of responsiveness to OW semaglutide treatment.

## Methods

### Patients

This is a single-center, retrospective, observational cohort study conducted at the Endocrinology and Diabetology outpatient clinic, Ospedale Maggiore della Carità, Novara, Italy. The study included patients with an established diagnosis of T2D, who underwent OW semaglutide treatment between September 2019 and May 2021 and were followed over 12 months. The study was notified to the Ethics Committee and performed in accordance with the current legislation on Observational Studies and the Declaration of Helsinki. Patients provided their written consent to process personal data, and the publication of the study results. For this study, patients previously diagnosed with T2D aged ≥ 18 years who were on a standard-care treatment regimen with oral antidiabetic drug (OAD) or insulin therapy were considered eligible for OW semaglutide if not on target with standard OAD, i.e. HbA1c ≥ 7% (≥ 53 mmol/mol), or if any of the following inclusion criteria was accomplished: HbA1c < 7% but intolerance towards ≥ 1 OAD other than GLP-1RA; previous CVE or high/very high CVR according to ESC 2019 guidelines [13]; estimated glomerular filtration rate (eGFR) between ≥ 15 and ≤ 30 ml/min/1.73 m^2^; BMI ≥ 35 kg/m^2^. Patients were not excluded if on other GLP-1 RA or dipeptidyl peptidase 4 inhibitors (DPP4i), which were then replaced by OW semaglutide upon enrollment. According to regulatory restrictions at the time of the study, gliflozins and short-acting insulins were withdrawn at study entry. Exclusion criteria included gestational diabetes mellitus and T1DM, previous or current diagnosis/family history of medullary thyroid carcinoma or multiple endocrine neoplasia, incomplete anthropometric and/or biochemical data at baseline and during follow-up visits. Patients were excluded from the study if showing poor compliance to OW semaglutide (fear of daily subcutaneous injection, elderly people unable to self-administer the drug, people suffering from senile dementia and/or neurodegenerative diseases) or if they were suffering from ESRD requiring dialysis or kidney disease unrelated to diabetes or hypertension.

### Study measures

Before enrollment, all patients were on stable OAD or insulin treatment for at least 3 months. At baseline, data collected for analysis included clinical history, antidiabetic drug history, anthropometric and biochemical parameters. Study measures included BMI, glucose, HbA1c, lipids, liver function tests, serum creatinine and microalbuminuria. Height was measured by the Harpenden stadiometer to the nearest mm with the subject head in Frankfurt plane and weight using electronic scale both taken in triplicate. Averaged BMI was calculated as body weight divided by squared height (kg/m^2^). The estimated glomerular filtrate rate (eGFR) was calculated with the MDRD [22] and CKD-EPI equation [23], yet kidney function was analyzed according to eGFR estimated by CKD-EPI, because MDRD equation has a low accuracy for an eGFR > 90 ml/min/1.73 m^2^ [22].

During treatment, sample data were evaluated as a whole and after dichotomization by categoric (gender, insulin therapy, previous CV events) and continuous variables (age, disease duration, baseline HbA1c, baseline BMI, baseline eGFR). Further, stratification by kidney function was performed according to eGFR CDK-EPI categories (< 60 ml/min, ≥ 60–89 ml/min, ≥ 90 ml/min).

Plasma glucose levels (mg/dl; 1 mg/dl: 0,05551 mmol/l) were measured by the gluco-oxidase colorimetric method (GLUCOFIX, by Menarini Diagnostici, Florence, Italy). Routine laboratory data included total cholesterol, high-density and low-density lipoprotein cholesterol, triglycerides, aspartate aminotransferase, alanine aminotransferase, gamma-glutamyl transpeptidase, measured by enzymatic methods (Roche Diagnostics, Mannheim, Germany). HbA1c levels were measured by high-performance liquid chromatography (HPLC), using a Variant machine (Biorad, Hercules, CA); intra- and inter-assay coefficients of variation are, respectively, lower than 0.6 and 1.6%. Linearity is excellent from 3.2 (11 mmol/mol) to 18.3% (177 mmol/mol). Serum creatinine levels were assessed with the enzymatic method of creatinine deamidase/GLDH (Advia Chemistry-Bayer).

Safety data were collected during the study period at each time visit.

### Statistical analysis

Data obtained from computerized medical record system in use at Italian diabetes centers (Smart Digital Clinic, Meteda, Rome, Italy) were used for the analysis. Data are expressed as mean ± standard error of the mean (SEM), and as absolute or percent delta variations compared to baseline. Distributions of continuous variables were examined for skewness and were logarithmically transformed as appropriate. Differences between values obtained at baseline and during the follow-up were calculated by two-tailed unpaired *t*-test or repeated measures ANOVA. Multivariate analysis of variance (MANOVA) was used to test the statistical significance of the effect of one or more independent variables including HbA1c variation, sex, age and duration of disease within the eGFR CDK-EPI categories (dependent variable). Correlations analyses were calculated with Pearson’s coefficient. Analysis of repeated measures was used to determine differences in subjects before and after treatment with OW semaglutide. As covariates, we assessed the effect of gender, age, BMI, disease duration, presence of diabetic complication/s, use of insulin, previous CV events, number of drugs for hypertension, use of ACE-inhibitors, angiotensin receptor blockers (ARBs), or diuretics (yes/no). A stepwise regression was used to determine the association of delta variations between HbA1c and study covariates, so as to define independent predictors of HbA1c response to OW semaglutide. Statistical significance was assumed at *p* < 0.05. The statistical analysis was performed with SPSS for Windows V.21.0 (SPSS Inc., Chicago, IL, USA).

## Results

### Study population and baseline clinical characteristics

**Fig. 1:**
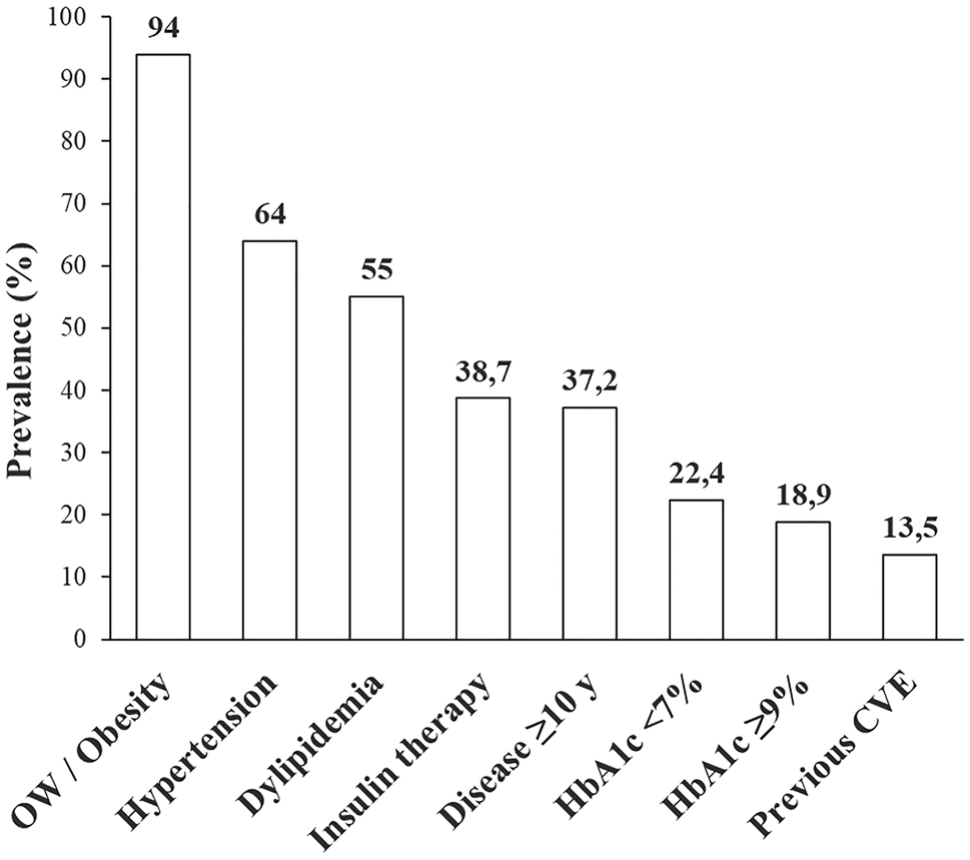
Baseline clinical features of TD2 patients included in the study. *OW* overweight, *CVD* cardiovascular disease

The original cohort of T2D patients receiving at least one prescription of OW semaglutide comprised 271 people, in 258 of whom a complete dataset allowed baseline analysis (Table 1 and Fig. 1). At study entry, 19 patients (7.3%) were naïve to any antidiabetic treatment, while OW semaglutide was added to 1 OAD in 136 (52.7%), to 2 OADs in 73 (28.2%), to 3 OADs in 11 cases (4.2%). Most but not all patients (84.5%) were GLP-1RA naïve. Biguanides (79.1%), followed by SGLT2i (20.1%), sulphonylureas (14.3%) and DPP4i (5.8%) were the non-insulin antidiabetic medications used in this cohort. Before OW semaglutide, metformin was used as single treatment in 74 cases (28.7%); long-acting insulin was overall used in 100 cases (38.7%) and as single treatment in 19 cases (7.3%).

**Table 1:** Demographic, anthropometric and metabolic variables in the population as a whole and after stratification by gender

Variables	Overall population	Males	Females	*p*-value
Sample (%)	258	151 (58.5)	107 (41.5)	0.2
Age (years)	60.4 ± 0.5	61.0 ± 0.6	59.5 ± 0.9	0.2
Disease duration (years)	8.6 ± 0.4	8.9 ± 0.6	8.2 ± 0.7	0.4
Weight (kg)	92.5 ± 1.1	94.7 ± 1.3	89.4 ± 1.8	0.2
BMI (kg/m^2^)	32.7 ± 0.4	**31.3 ± 0.4**	**34.7 ± 0.7**	** < 0.0001**
Systolic blood pressure (mmHg)	145 ± 1.2	145.8 ± 1.7	144.8 ± 1.8	0.7
Diastolic blood pressure (mmHg)	86.0 ± 0.6	86.7 ± 0.8	84.5 ± 0.9	0.1
HbA1c (%)	8.0 ± 0.6	8.0 ± 0.7	8.0 ± 0.7	0.2
Glucose (mg/dL)	163.7 ± 3.3	163.3 ± 3.8	164.2 ± 6.1	0.9
Microalbuminuria (mg/L)	30.4 ± 3.7	**38.2 ± 5.6**	**19.3 ± 3.3**	**0.05**
Creatinine (mg/dL)	0.9 ± 0.3	**1.00 ± 0.02**	**0.72 ± 0.02**	**0.0001**
eGFR (mL/min)	82.2 ± 1.9	**68.3 ± 1.51**	**102 ± 3.3**	**0.0001**
Total CHO (mg/dL)	173.0 ± 2.4	**165.2 ± 3.1**	**183.7 ± 3.4**	**0.0001**
LDL CHO (mg/dL)	91.0 ± 2.1	**86.8 ± 2.7**	**96.8 ± 3.4**	**0.02**
HDL CHO (mg/dL)	48.0 ± 1.2	**44.7 ± 1.4**	**53.6 ± 1.8**	**0.0001**
TG (mg/dL)	167.0 ± 5.4	170.5 ± 7.8	162.7 ± 6.9	0.9
AST (UI/L)	24.0 ± 0.6	24.6 ± 0.7	22.4 ± 0.9	0.3
ALT (UI/L)	30.5 ± 1.2	32.5 ± 1.2	27.7 ± 1.9	0.04
GGT (UI/L)	41.0 ± 2.4	44.1 ± 2.4	36.3 ± 3.4	0.1
Urate (mg/dL)	5.5 ± 0.12	**5.8 ± 0.1**	**5.2 ± 0.2**	**0.02**
Antidiabetic therapy				
Metformin	204 (79.1)	121 (80.1)	83 (77.6)	0.6
Sulfonylureas	37 (14.3)	17 (11.3)	20 (18.7)	0.09
DPP4-i	15 (5.8)	11 (7.3)	4 (3.7)	0.2
SGLT2-i	52 (20.1)	36 (23.8)	16 (15.0)	0.08
Insulin	101 (39.1)	62 (41.1)	39 (36.4)	0.4
Diabetes complications				
Nephropathy	22 (8.5)	**18 (11.9)**	**4 (3.7)**	**0.02**
Retinopathy	12 (4.7)	6 (4.0)	6 (5.6)	0.5
Stroke	6 (2.3)	3 (2.0)	3 (2.8)	0.7
CHD	30 (11.6)	**26 (17.2)**	**4 (3.7)**	**0.0009**
Peripheral arterial disease	30 (11.6)	21 (13.9)	9 (8.4)	0.2
Neuropathy	8 (3.1)	6 (4.0)	2 (1.9)	0.3
Concomitant therapies				
Anti-hypertensive drugs	169 (65.5)	101 (66.9)	68 (63.6)	0.6
Lipid lowering drugs	142 (55.0)	86 (57.0)	56 (52.3)	0.5
Cardiovascular events	35 (13.6)	**27 (17.9)**	**8 (7.5)**	**0.02**

Data are shown as mean ± SEM. *P* value is shown for gender-based analysis. Significant differences are shown in bold characters

*BMI* body mass index, *CHO* cholesterol, *TG* triglycerides, *AST* aspartate aminotransferase, *ALT* alanine aminotransferase, *GGT* gamma-glutamyl transferase, *DPP4-i* dipeptidyl peptidase-4 inhibitor, *SGLT2-i* sodium-glucose cotransporter-2 inhibitors, *CHD* coronary heart disease

Kidney function, as assessed by eGFR CDK-EPI, was ≤ 30 ml/min in 1 case, > 30–59 ml/min in 62 (24.1%), ≥ 60–89 ml/min in 115 (44.4%) and ≥ 90 ml/min in 80 patients (30.9%).

At baseline, there were no differences in the tested variables between genders but for BMI, this being unexpectedly higher in females than males, as well as microalbuminuria, creatinine, lipids, ALT and urate levels (Table 1).

### Outcomes of OW semaglutide treatment

Of the 258 consecutive patients, 154 completed the 6-month and 56 completed the 12-month follow-up. The samples scrutinized at the different timepoints hence reflected the patients consecutively enrolled until the study end with no dropouts. Following the standard dose-escalation protocol [16], OW semaglutide was given at the prefixed dose of 0.5 mg weekly in all cases for the first 6 months, and 1.0 mg weekly in 21.8% of the 12-month completers.

Improvements in glycemic control were significant in terms of HbA1c and glucose levels reduction (*p* < 0.0001 for both) both at 6 and 12 months (Table 2). HbA1c levels decreased by − 1.02 ± 0.1% after 6 months and − 1.1 ± 0.1% after 12 months of OW semaglutide. Target HbA1c values < 7% were achieved in 61% of 6-month completers and 57% of 12-month completers. The residual number of cases with HbA1c ≥ 9% by the study-end was low (5.3% vs 18.9% at baseline). After 6 months, HbA1c response to treatment was non-significantly different between GLP-1RA naïve patients compared to those previously exposed to any GLP-1RA (− 1.1 ± 0.1 vs − 0.64 ± 0.2%), while the study sample was too small for comparison at 12 months. Compared to study entry, the proportion of insulin users slightly decreased by 6 months (56 vs 53 cases: withdrawn in 10, newly added in 7, and continued in 46 cases) and 12 months (19 vs 16 cases: withdrawn in 6, newly added in 3, and continued in 13 cases).

**Table 2:** Demographic, anthropometric and metabolic variables in the T2D population during OW semaglutide treatment

Variables	Basal (*N* = 258)	6 months (*N* = 154)	12 months (*N* = 56)	Repeated measures ANOVA
HbA1c (%)	**8.0 ± 0.6**	**6.9 ± 0.1**^**$**^	**6.9 ± 0.1**^**$**^	** < 0.0001**
Glucose (mg/dL)	**163.7 ± 3.3**	**130.2 ± 3.1**^$^	**128.8 ± 4.3**^**$**^	** < 0.0001**
Weight (kg)	**92.5 ± 1.1**	**89.9 ± 1.5**^$^	**87.2 ± 2.5**^**$**^	**0.001**
BMI (kg/m^2^)	**32.7 ± 0.4**	**31.9 ± 0.5**^**$**^	**30.9 ± 0.8**^**$**^	** < 0.0001**
Total CHO (mg/dL)	**173.0 ± 2.4**	**150.2 ± 3.4**^**$**^	160.5 ± 6.6	0.09
LDL CHO (mg/dL)	**91.0 ± 2.1**	**77.4 ± 2.9**^**$**^	**85.4 ± 55.2**	0.2
HDL CHO (mg/dL)	**48.0 ± 1.2**	**44.1 ± 1.2**^**ϕ**^	50.5 ± 2.7	0.4
TG (mg/dL)	**167.0 ± 5.4**	**139.0 ± 5.9**^**θ**^	134.0 ± 7.5	0.4
Microalbuminuria (mg/L)	30.4 ± 3.7	22.2 ± 4.3	15.5 ± 1.88	0.08
Creatinine (mg/dL)	0.86 ± 0.3	0.85 ± 0.02	0.86 ± 0.03	0.6
eGFR (mL/min)	82.2 ± 1.9	80.7 ± 2.4	81.5 ± 4.2	0.2
AST (UI/L)	24.0 ± 1.3	21.9 ± 1.5	25.7 ± 2.7	0.6
ALT (UI/L)	30.5 ± 1.2	25.5 ± 1.3	25.7 ± 2.7	0.3
GGT (UI/L)	**41.0 ± 2.4**	**28.2 ± 2.2**^**$**^	**27.9 ± 4.5**^ϕ^	0.06
Urate (mg/dL)	6.0 ± 0.12	5.4 ± 0.3	5.3 ± 0.3	0.4
SBP (mmHg)	**145.0 ± 1.2**	**141.5 ± 1.7**^**θ**^	**143.1 ± 2.3**^**θ**^	**0.013**
DBP (mmHg)	86.0 ± 0.6	83.8 ± 1.1	83.5 ± 1.1	0.1

Data are shown as mean ± SEM. The comparative analysis was performed at 6 and 12 months vs baseline using the corresponding index cases

*BMI* body mass index, *SBP* systolic blood pressure, *DBP* diastolic blood pressure, *CHO* cholesterol, *TG* triglycerides, *AST* aspartate aminotransferase, *ALT* alanine aminotransferase, *GGT* gamma-glutamyl transferase

^θ^*p* < 0.05; ^ϕ^*p* < 0.01; ^$^*p* < 0.001 vs baseline. Significant differences are shown in bold characters

OW semaglutide treatment determined weight loss in 73.5% of patients after 6 months (ranging between − 0.5 and − 19 kg) and 78.1% after 12 months (ranging between − 0.2 and − 26 kg). The overall reduction in bodyweight was significant both in terms of body mass and BMI values (Table 2). Compared to baseline, weight loss was ≥ 5% in 21.2% and 25.4% of cases after 6 and 12 months, respectively, and ≥ 10% in 6.8% and 18.2% of cases after 6 and 12 months of OW semaglutide treatment, respectively.

The lipid profile improved during the first 6 months of treatment in terms of total cholesterol, LDL cholesterol, HDL cholesterol and triglycerides when compared to baseline (Table 2). We did not record changes in kidney function, both in terms of eGFR and microalbuminuria. Likewise, no change was recorded in any liver function tests but for a reduction in GGT values (Table 2).

### Subgroup analyses

**Fig. 2:**
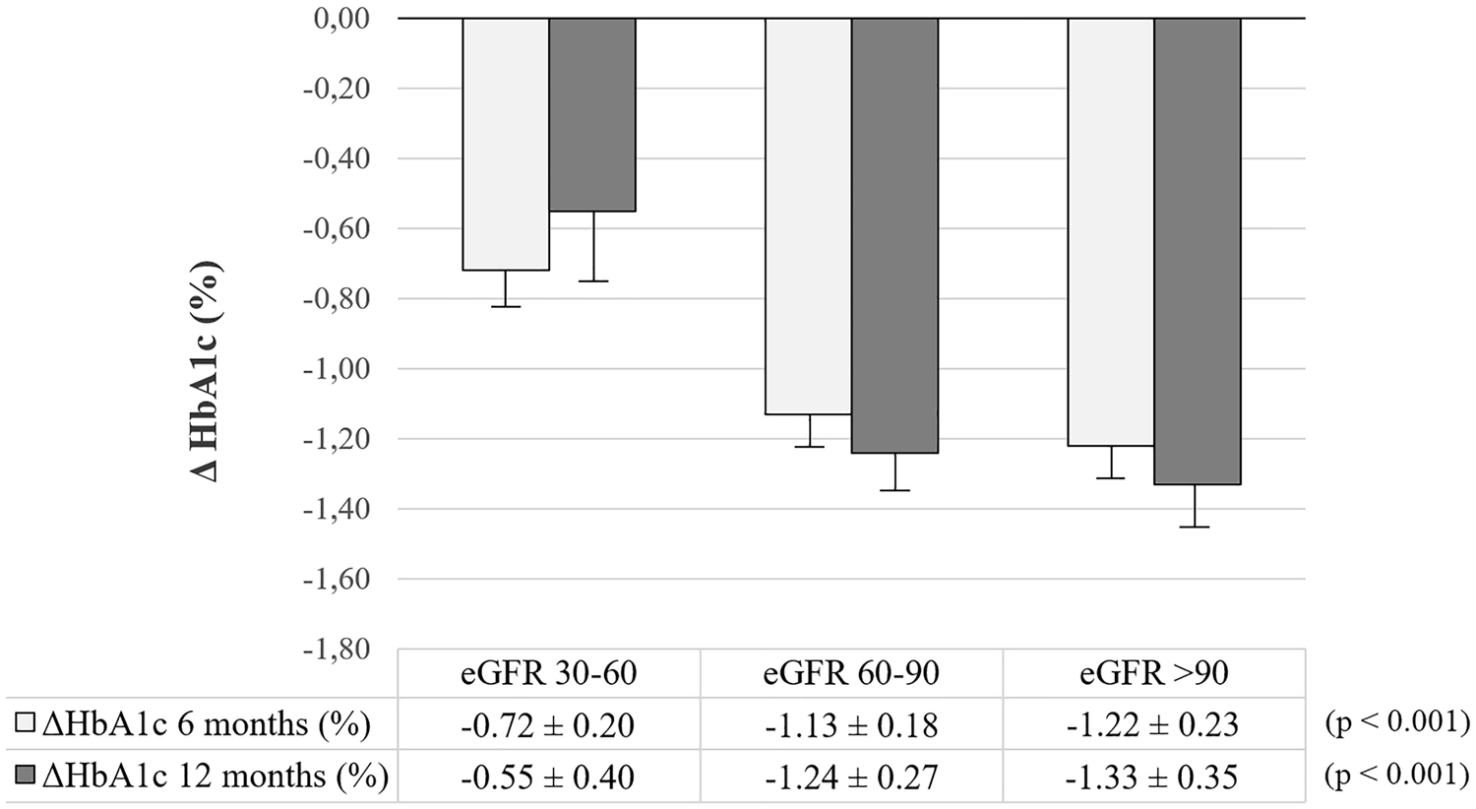
Changes in HbA1c levels after 6 and 12 months of treatment with OW semaglutide in T2D patients stratified by glomerular filtration rate (eGFR CDK-EPI) uncontrolled for age, gender and disease duration. For a description of the results of the multivariate-controlled analysis see the text

Subgroup analyses were conducted to test HbA1c responsiveness to OW semaglutide according to age, gender, baseline HbA1c and BMI, insulin, complications and previous CVE. We found no divergence in HbA1c response across subcategories except for patients with higher baseline HbA1c after 6 and 12 months, patients with higher disease duration after 6 months, and patients with previous CVE as compared to lower-end counterparts (Table 3). Stratification of patients according to the GFR CDK-EPI categories highlighted a significantly greater HbA1c response to OW semaglutide along with improving kidney filtration (Fig. 2). However, this difference in HbA1c response was lost after controlling for age, sex, BMI and duration of disease.

**Table 3:** HbA1c response (%) to OW semaglutide in subgroups

Variables	OW semaglutide			
	6 months		12 months	
	Bottom	Top	Bottom	Top
Age (median, 61 y)	− 1.2 ± 0.2	− 0.9 ± 0.1	− 1.0 ± 0.3	− 1.1 ± 0.2
Gender (0, females; 1, males)	− 1.1 ± − 1.0	− 1.0 ± 0.1	− 1.3 ± 0.3	− 1.0 ± 0.2
Disease duration (median, 8.1 y)	− **1.3 ± 0.2**	− **0.8 ± 0.1**^**θ**^	− 1.2 ± 0.3	− 1.0 ± 0.2
HbA1c (median, 7.9%)	− **0.4 ± 0.1**	− **1.8 ± 0.2**^**$**^	− **0.2 ± 0.2**	− **2.2 ± 0.2**^**$**^
BMI (median, 31.8 kg/m^2^)	− 0.8 ± 0.1	− 1.2 ± 0.2	− 1.1 ± 0.2	− 1.1 ± 0.3
Insulin treatment (0, no; 1, yes)	− 1.2 ± 0.1	− 0.8 ± 0.2	− 1.3 ± 0.2	− 0.7 ± 0.3
Complications (0, no; 1, yes)	− 1.2 ± 0.1	− 0.9 ± 0.5	− 1.2 ± 0.2	− 0.5 ± 0.6
Previous CVE (0, no; 1, yes)	− **1.1 ± 0.1**	− **0.4 ± 0.3**^**θ**^	− **1.2 ± 0.2**	− **0.3 ± 0.3**^**θ**^

^θ^*p* < 0.05; ^$^*p* < 0.001 top vs bottom end. Significant differences are shown in bold characters

*BMI* body mass index, *CVE* cardiovascular event

### Correlation and multivariate regression analyses

**Fig. 3:**
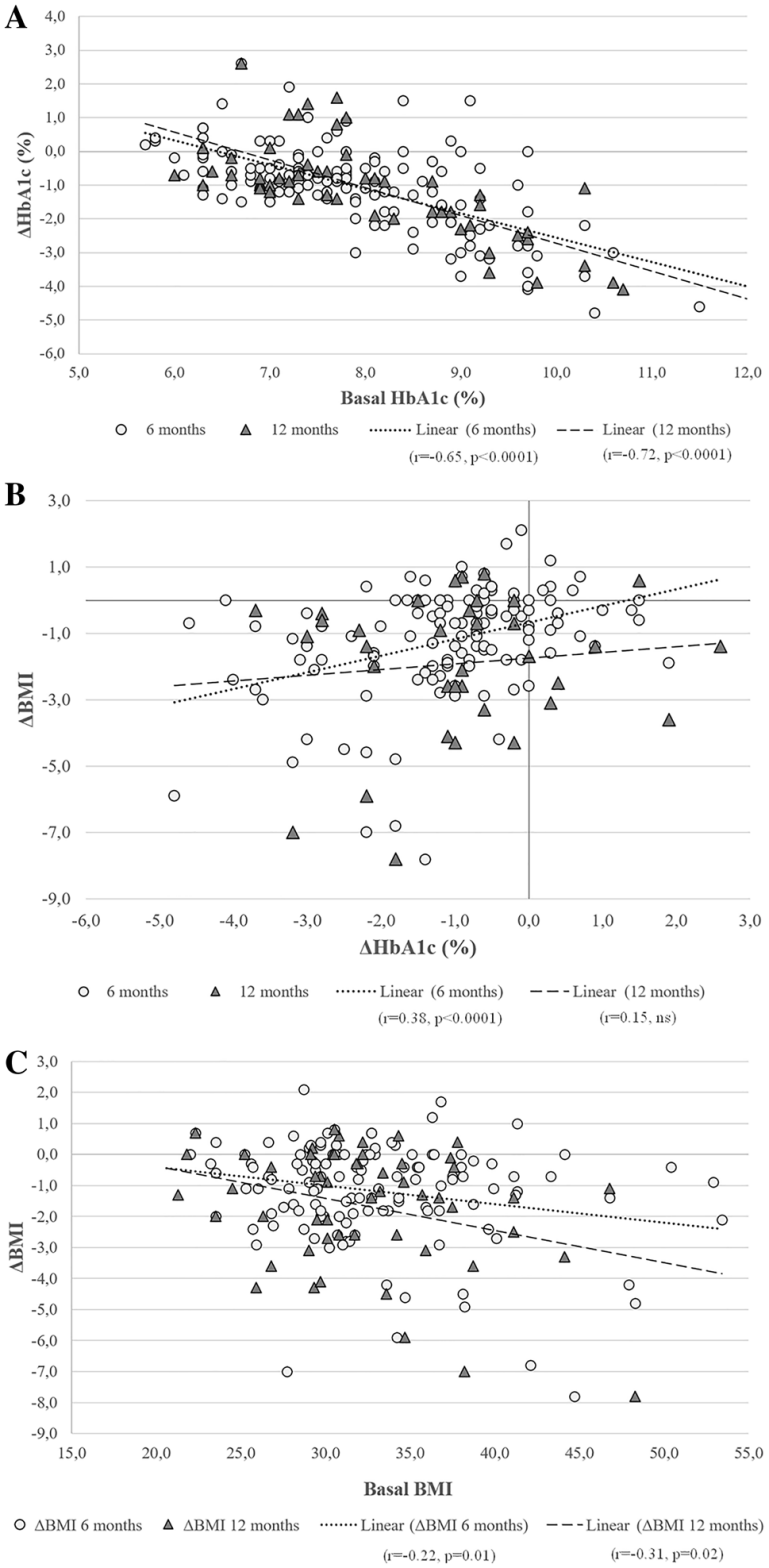
Correlation analyses between baseline HbA1c values and delta HbA1c reduction after 6 and 12 months of treatment with OW semaglutide (panel **A**); baseline BMI values and delta BMI reduction after 6 and 12 months of treatment with OW semaglutide (panel **B**); delta HbA1c and delta BMI variations after 6 and 12 months of treatment with OW semaglutide (panel **C**). Individual correlation coefficients and significance are reported in the figure

A wide number of associations were tested to assess correlates of HbA1c response to OW semaglutide. It should be noted that signs of association reflected deltas or absolute variables. Delta HbA1c response to OW semaglutide was well correlated with baseline HbA1c levels both after 6 and 12 months of the study (*r* = − 0.65 and *r* = − 0.72, respectively; *p* < 0.0001 for both) (Fig. 3A). Conversely, an association was observed between delta HbA1c and BMI variations only after 6 months (*r* = 0.38, *p* < 0.0001) (Fig. 3B). In turn, BMI deltas correlated with baseline BMIs after 6 months (*r* = − 0.22, *p* = 0.01) and after 12 months (*r* = − 0.31, *p* = 0.02) (Fig. 3C). At both time-points, associations related delta HbA1c to percent variations in blood glucose (*r* = 0.55 and *r* = 0.67; *p* < 0.0001 for both), triglycerides (*r* = 0.29, *p* = 0.002; and *r* = 0.45, *p* < 0.0001), and GGT levels (*r* = 0.33, *p* = 0.002; and *r* = 0.36, *p* < 0.05). Associations were only significant after 6 months between delta HbA1c and disease duration (*r* = 0.22, *p* < 0.05) as well as ALT variation (*r* = 0.43, *p* < 0.0001).

At stepwise multivariable regression analysis (Table 4), the most significant predictors of delta HbA1c variations after 6 months of OW semaglutide treatment included baseline HbA1c values (*β* =  − 0.59, *p* < 0.0001), delta BMI variation (*β* = 0.24, *p* < 0.0001) and disease duration (*β* = 0.22, *p* < 0.001). The adjusted *R*^2^ for the model was 0.52.

**Table 4:** Stepwise multivariable regression analysis showing independent predictors of delta HbA1c variations after 6 months of OW semaglutide treatment

Dependent variables	Model	Independent variables	Unstandardized coefficients		Standardized coefficient	*t*	*p*-value
			*B*	SE	Beta		
ΔHbA1c after 6 months	1	Constant	4.57	0.62	–	7.41	< 0.0001
		Basal HbA1c	− 0.71	0.08	− 0.64	− 9.21	< 0.0001
	2	Constant	4.39	0.58	−	7.54	< 0.0001
		Basal HbA1c	− 0.65	0.07	− 0.59	− 8.89	< 0.0001
		ΔBMI after 6 months	0.21	0.05	0.27	4.09	< 0.0001
	3	Constant	3.97	0.57	−	6.94	< 0.0001
		Basal HbA1c	− 0.65	0.07	− 0.59	− 9.22	< 0.0001
		ΔBMI after 6 months	0.19	0.05	0.25	3.81	< 0.0001
		Duration of disease	0.04	0.11	0.22	3.41	0.001
ΔHbA1c after 12 months	1	Constant	5.55	0.89	–	6.22	< 0.0001
		Basal HbA1c	− 0.83	0.11	− 0.72	− 7.53	< 0.0001

Excluded variables: model 1: basal BMI, ΔBMI, age, duration of disease, sex, basal eGFR, complications, previous CVE; model 2: basal BMI, age, duration of disease, sex, basal eGFR, complications, previous CVE; model 3: basal BMI, age, sex, basal eGFR, complications, previous CVE

*BMI* body mass index, *CVE* cardiovascular event

At 12 months, baseline HbA1c levels were the only predictors of delta HbA1c variation (*β* =  − 0.72, *p* < 0.0001) (Table 4), with an adjusted R^2^ for the model of 0.51.

### Safety data

A total of 39 out of 258 patients (15.1%) receiving at least one dose of OW semaglutide withdrew from treatment. OW semaglutide was discontinued in 29 cases (11.2%) for gastrointestinal intolerance occurring within few weeks since therapy commencement (nausea and vomiting in 16, diarrhea or constipation in 7, abdominal cramps in 2 and malaise in 1 patient), while treatment was discontinued due to therapeutic inefficacy in 9 patients and pregnancy in 1 case. In 3 patients (0.1%), OW semaglutide was withdrawn for poor compliance. In 9 more patients, dose increment from 0.5 mg to 1.0 mg weekly was constrained due to gastrointestinal intolerance occurring during dose titration. However, gastrointestinal side effects did not appear to contribute to the reductions in HbA1c and/or body weight.

## Discussion

RWE studies on GLP-1RAs have provided extensive evidence that OW semaglutide reduces HbA1c and body weight irrespective of previous GLP-1 RA use, with potentially higher persistence than other GLP-1 RAs [24–43]. Our results show that OW semaglutide induced significant reduction in mean HbA1c independent of age, gender, insulin therapy, prior history of GLP-1RA use and diabetic complications. Baseline HbA1c levels, disease duration and previous CVE appeared to influence HbA1c responsiveness to OW semaglutide. Our study also adds evidence that HbA1c response only initially reflects a legacy involving bodyweight changes, and hints at a potential regulatory role of kidney function on OW semaglutide effectiveness.

GLP‐1 analogs are recommended as add‐on therapy to metformin and other OADs and as first injectable therapy in preference to insulin [44, 45]. Among different GLP-1RA options, semaglutide occupies a prominent place in treating T2D patients at high CVD and DKD risk [46, 47]. Data from clinical trials highlighted the effectiveness and potency of OW semaglutide even in comparison to other same-class agonists [48–50]. Confirming the vast interest aroused by semaglutide in the treatment of diabetes and obesity, an oral formulation semaglutide has been recently introduced [51], which is particularly useful for patients who dislike injections, and major medicines regulatory agencies have recently granted approval for the use of OW semaglutide in the treatment of obesity [52].

The patient population herein scheduled to receive OW semaglutide was moderately aged, averagely complicated, and predominantly obese. Our results show that OW semaglutide was associated with clinically and statistically significant reduction in mean HbA1c, regardless of age, gender, insulin therapy, prior history of GLP-1RA use and diabetic complications. HbA1c decreased on average by 1.0–1.1%, and the magnitude of response was greater in patients with higher baseline HbA1c levels. Target HbA1c values < 7% were achieved in 61% and 57% of cases after 6 and 12 months, respectively. Our results seem comparable to those of other RWE studies. While the SUSTAIN studies reported a mean HbA1c reduction from baseline of 1.0–1.5% and HbA1c < 7% in 68.7–82.8% of cases after 6–12 months of treatment [53], recent retrospective observational studies documented a mean HbA1c reduction of 0.8–1.2% from baseline, with rates of patients achieving HbA1c < 7% ranging between 48.6–53% [24, 26]. Further, the number of patients with HbA1c persisting at ≥ 9% values by the end of treatment was 5.3%, a marginal figure that is comparable to rates recorded in registry-based studies [24, 26].

In addition to its anti-hyperglycemic effects, OW semaglutide outstands for its ability to reduce body weight thus aiding the global metabolic control. As a proof of this, we observed significant changes in body weight, with an average reduction of 3.1 kg after 6 months and 4.3 kg after 12 months as compared to baseline. Collectively, weight loss was ≥ 5% in 21 and 25.4% after 6 months and was ≥ 10% in 6.8 and 18.2% of cases after 12 months of treatment. Recent RWE have shown similar effects on weight [25, 32]. It is worth noting that a strong association related the variation between HbA1c and weight after 6 but not at the 12 months follow-up. On one hand, this implies a key role for OW semaglutide in controlling the whole metabolic spectrum while, on the other, it seems to disentangle the anti-hyperglycemic and weight-reducing effects of OW semaglutide over time.

Based on previous data from our group [54], a goal of our study was also the evaluation of renal function in terms of changes in eGFR and microalbuminuria. Current data showed no effect of OW semaglutide on these parameters, although a response trend emerged for microalbuminuria. Others reported similar results [25, 32] and post-hoc analyses suggested that OW semaglutide can initially decrease eGFR while promoting reductions in UACR [55]. While a conservative analysis of data stratified by kidney function hinted at a potential enhancing effect of kidney filtration rate on HbA1c response after 6 and 12 months, significance was lost when age, gender and disease duration were controlled for. We are therefore inclined to speculate on the significance of better kidney health as a function of younger age, gender difference, shorter duration of disease and overall more preserved β-cell function. As such, older patients with a long history of disease and previously exposed to insulin-secretagogues may show lower response to drugs other than insulin [56]. The open question remains if semaglutide can promote recovery of damaged β-cell function in case of long-term use of insulin-secretagogues, which may delay needs for insulin.

In insulin users, initiation of OW semaglutide led to a reduction in HbA1c and body as significant and comparable as in non-insulin users. Like another RWE analysis [57], we recorded a marginal insulin discontinuation rate. Yet, the insulin withdrawal rate was greater the newly add-on rate by the study end, suggesting the support provided by OW semaglutide in insulin de-intensification. It has been previously shown that patients on OADs are significantly more likely to reach target HbA1c and lose body weight when treatment is intensified with GLP-1 RA as compared to either OADs or insulin [53, 58–60]. An analysis capturing costs, mortality, and quality of life found similar results [61]. Together, these data confirm guidelines recommendations [45] to consider GLP1-RA therapy as an injective alternative to insulin therapy in patients not achieving treatment targets, allowing extended metabolic control associated with cardio-protection and weight loss, without the risks associated with hypoglycemia and possible weight gain deriving from the use of insulin.

Semaglutide was generally well-tolerated, though a lower proportion of persons experiencing side effects (18%) compared to the clinical studies were noted and this was predominated by gastrointestinal side effects, such as nausea, diarrhea or constipation, and abdominal cramps. No patient was admitted to hospital due to pancreatitis or for any other reason. The figure reported here is higher than that seen in the SUSTAIN-6 trial (13.1%) and some RWE studies [29, 32], while others reported rates similar to us [26]. Our study was not designed to assess hypoglycemic episodes, so we cannot draw conclusions on this issue.

This study presents limitations that are typical of retrospective observational studies, such as the lack of comparators and randomization, the access to utilization, and cost of health resources. Moreover, the low number of patients at the 12-month follow-up may hamper the conclusions of our analysis. It should also be acknowledged that the 1.0 mg dose was prescribed to only 22% of cases, implying that up-titration was incomplete in most cases possibly due to the choice of clinicians, concomitant therapies of the population sample, and underlying intentions to limit occurrence of side effects. Nevertheless, the effect of treatment on HbA1c and weight was similar to that reported in most RWE studies [24–31]. Points of potential strength of the study are constituted by the wide characterization of our cohort including 102 different variables and the broad representativeness of our patient population. Hence, this study sheds light on the impact of the different clinical and disease characteristics of T2D patients have on the efficacy and safety of OW semaglutide, which can help clinical decision‐making and individualization of treatment. Further real-world studies to evaluate semaglutide adherence and acceptability would be important and of clinical interest.

## Data Availability

The datasets generated during and/or analyzed during the current study are available from the corresponding author on reasonable request.
